# Nonequilibrium Magnetic Response of Anisotropic Superparamagnetic Nanoparticles and Possible Artifacts in Magnetic Particle Imaging

**DOI:** 10.1371/journal.pone.0118156

**Published:** 2015-03-16

**Authors:** Hiroaki Mamiya, Balachandran Jeyadevan

**Affiliations:** 1 National Institute for Materials Science, Tsukuba, 305-0047, Japan; 2 The University of Shiga Prefecture, Hikone, 522-8533, Japan; Glasgow University, UNITED KINGDOM

## Abstract

Magnetic responses of superparamagnetic nanoparticles to high-frequency AC magnetic fields with sufficiently large amplitudes are numerically simulated to exactly clarify the phenomena occurring in magnetic particle imaging. When the magnetic anisotropy energy inevitable in actual nanoparticles is taken into account in considering the magnetic potential, larger nanoparticles exhibit a delayed response to alternations of the magnetic fields. This kind of delay is rather remarkable in the lower-amplitude range of the field, where the assistance by the Zeeman energy to thermally activated magnetization reversal is insufficient. In some cases, a sign inversion of the third-order harmonic response was found to occur at some specific amplitude, despite the lack in DC bias magnetic field strength. Considering the attenuation of the AC magnetic field generated in the human body, it is possible that the phases of the signals from nanoparticles deep inside the body and those near the body surface are completely different. This may lead to artifacts in the reconstructed image. Furthermore, when the magnetic/thermal torque-driven rotation of the anisotropic nanoparticles as well as the magnetic anisotropy energy are taken into account, the simulated results show that, once the easy axes are aligned toward the direction of the DC bias magnetic field, it takes time to randomize them at the field-free point. During this relaxation, the third-order harmonic response depends highly upon the history of the magnetic field. This is because non-linearity of the anhysteretic magnetization curve for the superparamagnetic nanoparticles varies with the orientations of the easy axes. This history dependence may also lead to another artifact in magnetic particle imaging, when the scanning of the field-free point is faster than the Brownian relaxations.

## Introduction

Superparamagnetic nanoparticles as well as positron-emitting radionuclides rarely exist naturally in the human body and, furthermore, not only gamma rays emitted in positron annihilations but also magnetic flux generated by the nanoparticles can easily penetrate the human body. These facts demonstrate the great potential of magnetic particle imaging (MPI) as an alternative for the existing imaging method of positron emission tomography [1–6]. Using MPI for diagnosing small metastases involves creating a preferential accumulation of superparamagnetic nanoparticles in tumor tissue by conjugating nanoparticles with tumor-homing peptides or antibodies. An AC magnetic field is then irradiated on the nanoparticle-loaded cancer cells in the presence of an inhomogeneous DC bias magnetic field. Since only unsaturated superparamagnetic nanoparticles exhibit a nonlinear response to the AC magnetic field, the detection of higher-order harmonics of the electromotive force is an indication of the existence of tumor fragments at the DC bias field-free point. For practical use, the development of superparamagnetic nanoparticle tracers and irradiation apparatus has been promoted in recent years [4–9]. In particular, the use of nanoparticles with larger diameter has been intensively studied in the field of material science anticipating strong signal [4]. Additionally, efficient scanning of the field-free point is important for a rapid diagnosis from the viewpoint of systems engineering [7–9].

In contrast to the advances made in the fields of material science and systems engineering, an over simplified physical model is still in use in MPI [5,6]. Briefly, the detected signal is analyzed with the assumption that the nanoparticles are completely isotropic. In other words, the energy potential *U* is assumed to be expressed only by Zeeman energy for the magnetic field ***H***,

$$U\;=\;-\mu_{0}M_{\text{s}}e∙H,$$ (1)

where *μ*_0_ is the vacuum permeability, *M*_s_ is the magnitude of spontaneous magnetization of a uniformly magnetized single domain nanoparticle, *V* is the volume of the nanoparticle, and ***e*** is the unit vector along the direction of spontaneous magnetization. In this case, magnetization per unit volume of the tumor tissue, ***M***, is always parallel to ***H*** and its magnitude is expressed as *NM*_s_ *V·L*(*μ*_0_ *M*_s_ *V* ***e·H***/*k*_B_ *T*), where *N* is the particle number density, *L* is the Langevin function, *k*_B_ is the Boltzmann constant, and *T* is the temperature.

Here, we can recall that actual nanoparticles are more or less anisotropic [10]. In other words, the potential energy *U* depends upon the direction of ***e*** even at *H* = 0, and its local minima will block immediate magnetic responses, which may lead to non-equilibrium phenomena. Additionally, it should be remembered that the nanoparticles in a tumor tissue are, in most cases, rotatable (discussed later). This rotatability may be nontrivial for the magnetic response of the actual anisotropic nanoparticles, whereas the properties of completely isotropic ones are unaffected by such rotations. Therefore, the first part of this article reports the results of the simulation of the switching of the direction of ***e*** on *U* between the local minima, and discusses the effects of using larger nanoparticles upon the third-order harmonic of the electromotive force at the field-free point. The latter part of this article shows the results of the simulation of magnetic/thermal torque-driven rotation of anisotropic nanoparticles, and discuss how the high speed scanning of the field-free point affects their nonlinear magnetic response. Note that these simulations are performed only for superparamagnetic nanoparticles with Néel relaxations, because the detailed properties of the higher-order harmonics generated by the Brownian relaxations of the ferromagnetic nanoparticles have already been reported [11,12].

## Results and Discussion

### Sign inversion of the third-order harmonic component

**Fig 1:**
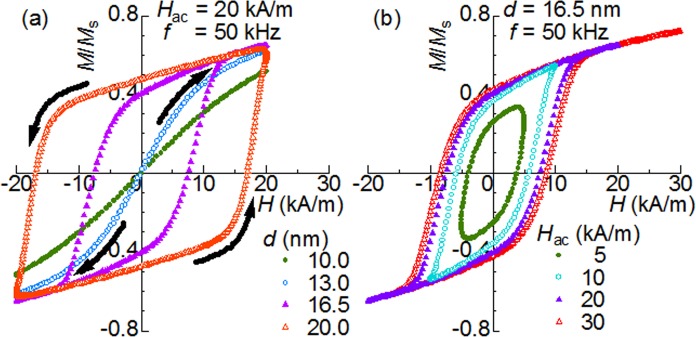
Dynamic magnetization curves. Dynamic magnetization curves calculated for the anisotropic nanoparticles with randomly-oriented easy axes when an AC magnetic field *H*_ac_·sin(2nπ*f*·*t*) is irradiated at a frequency *f* = 50 kHz. (a) Size *d*-dependence and (b) amplitude *H*_ac_-dependence. The arrows indicate the representative curvatures seen at *d* = 13 and 20 nm.

**Fig 2:**
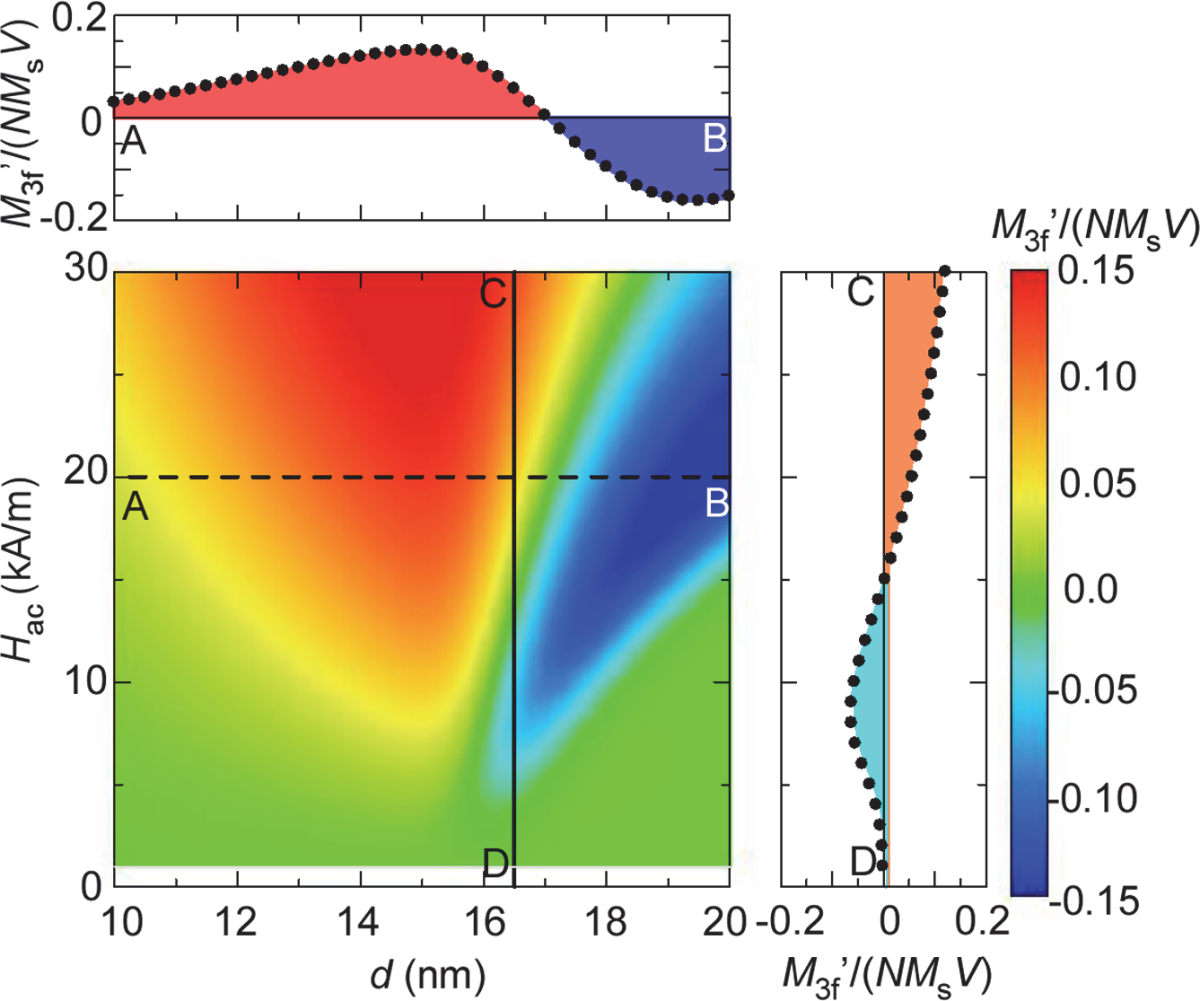
Contour plot of *M*_3f^′^_ for mono-disperse nanoparticles. Contour plot of the third-order harmonics of magnetization, *M*_3f^′^_, for mono-disperse nanoparticles with different sizes, *d*, in AC magnetic fields at various amplitudes, *H*_ac_, and *f* = 50 kHz. The upper and right-side panels show the cross-sections taken at the broken and solid lines, respectively.

Fig. 1(A) shows the dynamic magnetization curves calculated for the anisotropic nanoparticles with randomly-oriented easy axes for various particle diameters, *d*, when an AC magnetic field *H*_ac_·sin(2nπ*f*·*t*) with an amplitude *H*_ac_ = 20 kA/m is irradiated at a frequency *f* = 50 kHz. The physical quantities regarding the anisotropy of these nanoparticles are summarized in Table 1. There is no hysteresis found for *d* = 10 and 13 nm, which is consistent with the fact that the Néel relaxation time, ***τ***_N_ (shown in Table 1), is much shorter than the characteristic time of the field variation (2π*f*)^−1^ ∼ 3 μs. For *d* = 13 nm, the system is more easily magnetized compared with *d* = 10 nm. This is reasonable because the Zeeman energy given by Eq. (1) is greater for larger volumes. In addition, it is noteworthy that the magnetization curve for *d* = 13 nm is apparently convex at *H* > 0, while it is concave at *H* < 0, as indicated by the arrows. In other words, the nonlinear susceptibility *χ* ^2^ of *d* = 13 nm is negative and its magnitude is significantly larger than that of *d* = 10 nm, where the definition of *χ* ^2^ is given as *M* = *χ* ^0^ *H + χ* ^2^ *H* ^3^ *+ χ* ^4^ *H* ^5^ *+*… Therefore, while also regarding the Fourier series of *M*(*t*), $\sum_{\text{j}}\left[M_{\text{jf}}^{\prime }\text{sin}\left(2\text{j}\pi ft\right)-M_{\text{jf}}^{\prime \prime }\text{cos}\left(2\text{j}\pi ft\right)\right]$, this difference in the *χ* ^2^ between the two particle diameters indicates that the in-phase component of the third-order harmonics *M*_3f^′^_ at *d* = 13 nm is fairly large in comparison with *M*_3f^′^_ at *d* = 10 nm, because *M*_3f^′^_ is expressed as

$$M_{3f}^{\prime }\;=\;-\frac{1}{4}\chi_{2}H_{\text{ac}}^{3}\;-\frac{5}{16}\chi_{4}H_{\text{ac}}^{5}\;+\;\cdots$$ (2)

using the substitution *H = H*_ac_·sin(2nπ*f*·*t*). The upper cross-section view in Fig. 2 shows the size-dependence of *M*_3f^′^_, where the value of *M*_3f^′^_ seems to increase threefold as *d* increases from10 to 13 nm. The degree of this increase is weaker than is expected for the *χ* ^2^ = –(1/45)(*μ*_0_ *M*_s_ *V*/*k*_B_ *T*)^3^ ∞ *d* ^9^ term of the Langevin function. One of the reasons is that the increase in *V* affects the higher-order terms in Eq. (2) more remarkably.

**Table 1:** Physical quantities of magnetite nanoparticles of different sizes.

*d* (nm)	6.5	10	13	16.5	20	23
*K*_eff_ *V* (10^–21^J)	2.9	10.5	23.1	47.0	83.8	127.4
exp(*K*_eff_ *V/k*_B_ *T*)	2	1.3 × 10^1^	2.6 × 10^2^	8.5 × 10^4^	6.2 × 10^8^	2.3 × 10^13^
*τ*_N_	200 ps	1.3 ns	26 ns	8.5μs	62 ms	2300 s

Effective anisotropy energy *K*_eff_ *V*, Boltzmann factor exp(*K*_eff_ *V/k*_B_ *T*) and Néel relaxation time *τ*_N_ in *H* = 0 for magnetite nanoparticles with various effective diameters *d* = (6*V*/π)^1/3^, where *K*_eff_ is assumed to be 20 kJ/m^3^.

Before turning to the results for larger anisotropic nanoparticles, we note that *H*_ac_ is much smaller than the anisotropy field *H*_K_ of 72 kA/m for the assumed effective uniaxial anisotropy constant *K*_eff_ of 20 kJ/m^3^ (details in the Models section). In other words, these results correspond to minor loops in the conventional category. In Fig. 1(A), it can be seen that the magnetization curve for *d* = 16.5 nm exhibits an apparent hysteresis in the vicinity of zero magnetic field. The reason for this hysteresis is that the equilibration by the thermally-activated reversals cannot keep up with the variation of the AC magnetic field because ***τ***_N_ of 8.5 μs at *H* = 0 is longer than (2π*f*)^-1^ ∼ 3 μs (see Table 1.) On the other hand, the curve is anhysteretic in the range from 15–20 kA/m, although these fields are much lower than *H*_K_. A notable point is that ***τ***_N_ gets shorter as the barrier height is reduced by the Zeeman energy; consequently, it becomes shorter than (2π*f*)^- 1^ at 15 kA/m (250 ns in the case of ***n*** // ***H***, where ***n*** is the unit vector along the easy axis). Thus, the anhysteretic properties is reasonable in the field range. On the other hand, at *d* = 20 nm, ***τ***_N_ is still 7.4 μs at 20 kA/m. Certainly, no anhysteretic region can be seen in the results simulated for nanoparticles at this size. It is noteworthy that, as indicated by the arrows in Fig. 1(A), the shape of the magnetization curve for *d* = 20 nm seems concave at *H* > 0, while it is convex at *H* < 0, indicating that *χ* ^2^ for *d* = 20 nm has the opposite sign to *χ* ^2^ for *d* = 13 nm. This sign inversion exhibited by *M*_3f_ (∞ *χ* ^2^) can be clearly seen in the upper cross-section view in Fig. 2. To illustrate the size-dependence of *M*_3f_ at other amplitudes of *H*_ac_, a contour plot of *M*_3f_ is shown in the main panel of Fig. 2, where we find the same kind of the sign inversions occurring with smaller *d* particles when *H*_ac_ is lowered. This variation of the critical size with lowering *H*_ac_ is reasonable because the lower energy barriers of smaller particles are easily surmountable even with the lesser assist by the weak Zeeman energy at lower *H*_ac_. The *H*_ac_ dependence of *M*_3f_ exhibits an interesting property at the critical size, wherein the *M*_3f_ from nanoparticles with *d* = 16.5 nm is clearly negative at *H*_ac_ = 10 kA/m, is zero at *H*_ac_ = 15 kA/m, and is positive at *H*_ac_ = 20 kA/m, as shown in the right cross-section in Fig. 2. This can be attributed to the change of the predominant curvature from a hysteresis loop at lower fields to an anhysteretic curve at higher fields, as shown in Fig. 1(B).

At this stage, it is helpful to recall the inhomogeneity of *H*_ac_ using actual diagnoses. For example, *H*_ac_ is 30 kA/m at the center of the coil when an AC current of 1 kA is supplied to a 0.20 m-diameter coil with 6 turns. The value of *H*_ac_ weakens with distance from the coil, so that it becomes roughly 20, 15 and 10 kA/m at a distance of 0.07, 0.10, and 0.14 m on the central axis from the coil, respectively. This fact clearly indicates that, if the above-discussed nanoparticles (at *d* = 16.5 nm) are selected on the grounds that the signal is relatively large at the center of the coil, the sign of *M*_3f_ measured in the field-free points near the body surface will be different from those measured at points deep inside the body, while there will be no signal at all in the intermediate region, although the states of the accumulated superparamagnetic nanoparticles are identical with each other. Therefore, the image reconstruction process will cause artifacts unless we take into account this kind of sign inversion.

**Fig 3:**
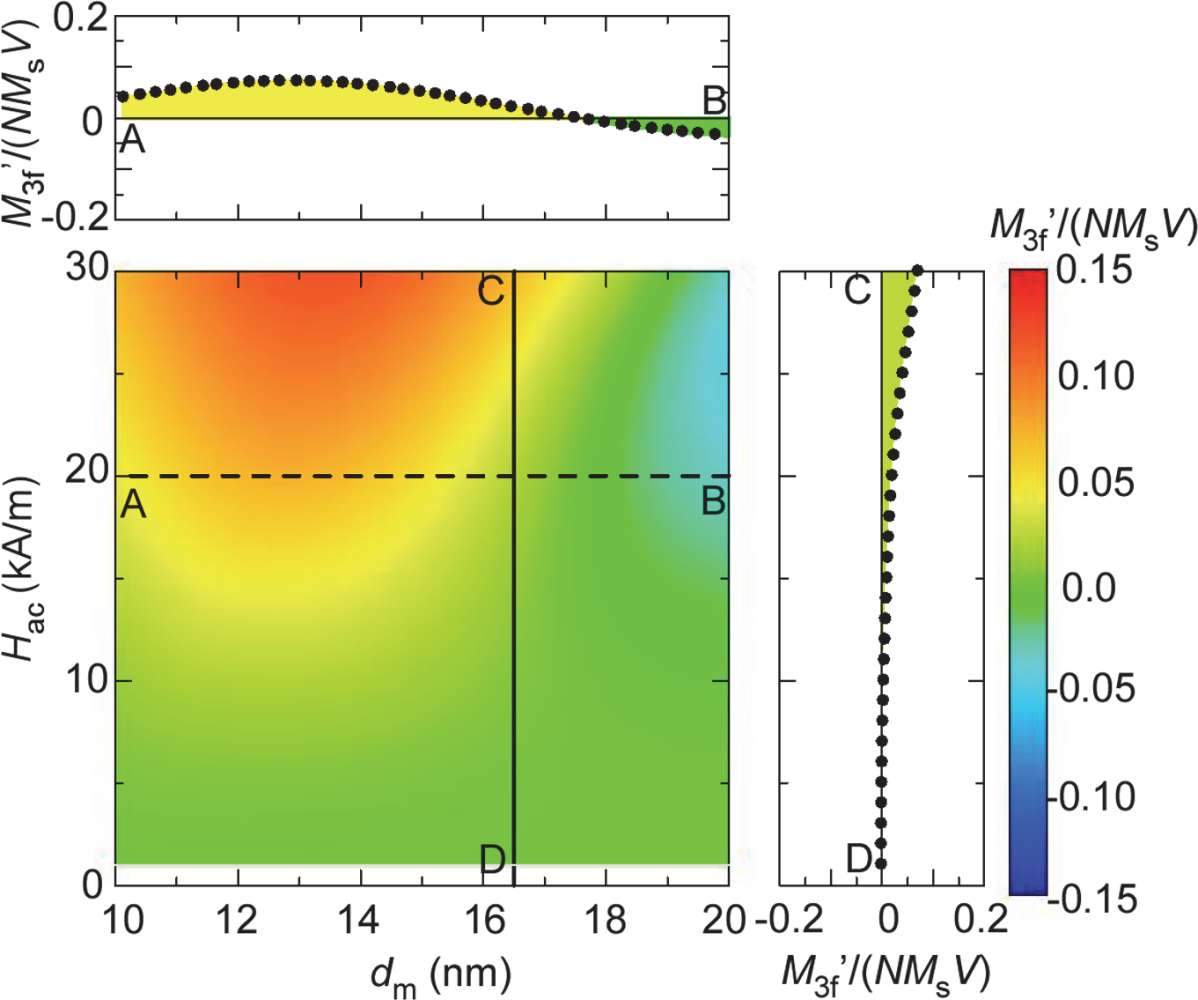
Contour plot of *M*_3f^′^_ for poly-disperse nanoparticles. Contour plot of the third-order harmonics of magnetization, *M*_3f^′^_, for poly-disperse nanoparticles with different mean sizes, *d*_m_, in AC magnetic fields at various amplitudes, *H*_ac_, and *f* = 50 kHz. The upper and right-side panels show the cross-sections taken at the broken and solid lines, respectively.

A major reason why little attention has been given to this phenomenon of sign inversion with variation in *H*_ac_ is that the nanoparticles used in previous studies are polydispersive. Fig. 3 shows a contour plot of *M*_3f_ for poly-disperse nanoparticles with a standard deviation *σ* = 0.2. In this case, the positive contributions from smaller particles offset the negative ones from larger particles, and the phenomena observed with mono-disperse nanoparticles is wiped out. Consequently, we can find no sign inversion in the cross-section from C to D in Fig. 3, and we did not need to consider such sign inversion. Mono-disperse nanoparticles are strong candidates for use in MPI in the near future, however, because the *M*_3f_ for poly-disperse nanoparticles is much weaker compared with that of mono-disperse nanoparticles (see again Figs. 2 and 3.) Therefore, it is important at this stage of mono-disperse nanoparticle selection to recall the sign inversion of *M*_3f_ with variation in *H*_ac_ and prepare nanoparticles with diameters slightly smaller than the size for which the sign of *M*_3f_ changes from positive for anhysteretic magnetization curves to negative for hysteretic ones. The desirable size for the case exemplified here is roughly 13–15 nm (< 16.5 nm) and the value of *M*_3f_ is highly positive at any *H*_ac_, as shown in Fig. 2.

### History dependence of the third-order harmonic component

**Fig 4:**
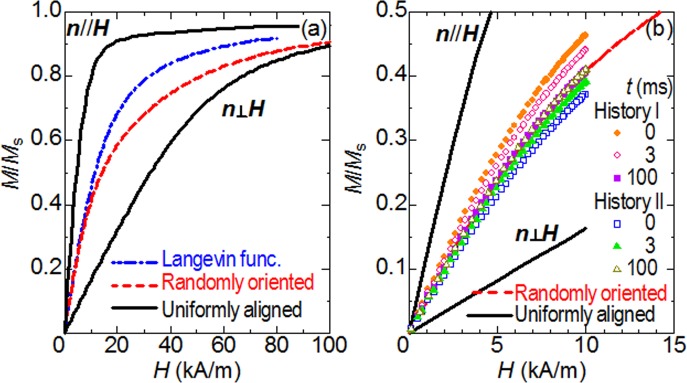
Magnetization curves calculated for anisotropic nanoparticles with a size of *d* = 13 nm. (A) Static magnetization curves calculated for nanoparticles whose easy axes are aligned at certain directions as well as for those with randomly-oriented easy axes. The dot-dashed line indicates the curve for the isotropic nanoparticles calculated using Langevin function. (B) Evolution of the dynamic magnetization curves with randomization of the directions of the easy axes for the field histories I and II (see also Fig. 5), where *H*_ac_ = 10 kA/m and *f* = 50 kHz.

The above section emphasizes the benefit of using superparamagnetic nanoparticles whose diameters are slightly smaller than the critical size. This raises the question whether the use of such particles could avoid any influence from the anisotropy of actual nanoparticles. Fig. 4(A) shows static magnetization curves calculated for the above-exemplified nanoparticles whose *d* = 13 nm, where the results for the cases when the easy axes are aligned at a certain direction are shown in addition to that of the case of randomly-oriented easy axes. We can see that, in the case where ***n*** // ***H***, the system is easily magnetized in comparison with the prediction expressed by the Langevin functions, while the magnetizing process is relatively hard for ***n*** ⊥ ***H***. To consider the reason for this difference, we note the Boltzmann factor of 260 and ***τ***_N_ of 26 ns for nanoparticles with *d* = 13 nm (Table 1). These values indicate that ***e*** is fairly bound around the two directions parallel/antiparallel to the easy axis ***n***, while the probabilities are instantly redistributed between them with changing Zeeman energy in the time scale of (2π*f*)^- 1^ ∼ 3 μs. This signifies that, for ***n*** // ***H***, the redistribution causes a significant increase in *M*(*t*) with increasing *H* because they occur from the direction antiparallel to ***H*** to the parallel direction. On the other hand, the reversals for ***n*** ⊥ ***H*** contributes little to the increase in *M*(*t*) because they occur from the direction perpendicular to ***H*** to the other perpendicular direction. For these reasons, the static magnetization curves of anisotropic superparamagnetic nanoparticles highly depend upon the orientations of the easy axes, in contrast to the prediction obtained using the Langevin functions [13–15]. Consequently, the nonlinearity for ***n*** // ***H*** is much greater than that for ***n*** ⊥ ***H***, as is apparently found in Fig. 4(A).

**Fig 5:**
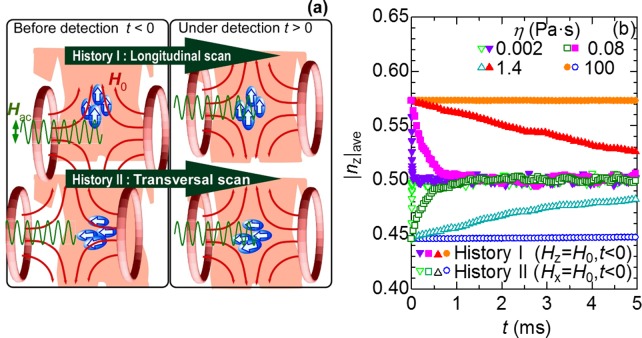
Maxwell coil pair scan schematics and calculated values for two field histories. (A) Typical scans of a DC bias field-free point in a Maxwell coil pair. In the longitudinal scan, the direction of the AC magnetic field, ***H***_ac_, is parallel to the DC bias magnetic field, ***H***_0_, applied just before the field-free point comes (history I), while ***H***_ac_ is perpendicular to ***H***_0_ in the transversal scan (history II). (B) Calculated variation of the average of the absolute value of the z-axis component of the unit vector along the easy axis ***n***, |*n*_z_|_ave_, for nanoparticles with *d* = 13 nm for the histories I and II after ***H***_0_ disappears at *t* = 0.

As sketched in Fig. 5(A), the history of the magnetic field while scanning the field-free point can be typified by the longitudinal scan (history I) and the transversal scan (history II). In the former case the AC magnetic field ***H***_ac_ = (0, 0, *H*_ac_·sin(2π*f*·*t*)) is parallel to the DC bias magnetic field ***H***_0_ = (0, 0, *H*_0_) that is applied just before the field-free point comes, while the ***H***_ac_ is perpendicular to ***H***_0_ = (*H*_0_, 0, 0) in the latter case. In this study, the magnetic responses of the above-discussed nanoparticles are simulated for these two model histories (details in the Models section). Fig. 5(B) shows the calculated variation of the average for the absolute value of the Z-axis component of ***n***, |*n*_z_|_ave_ for the nanoparticles with *d* = 13 nm after ***H***_0_ disappears at *t* = 0. The initial value of |*n*_z_|_ave_ is found to be larger than 0.5 for history I, which indicates that the easy axes, on average, are slightly oriented toward the z direction parallel to ***H***_0_ at *t* = 0. This is quite reasonable because there is competition between the magnetic torque that turns the easy axis toward the field direction and the Brownian torque that randomizes the directions of the easy axes. Also, we are certain that in history II, the easy axes are initially oriented toward the direction perpendicular to the z direction, because |*n*_z_|_ave_ is smaller than 0.5 at *t* = 0. When considering their relaxations, we see that all of the |*n*_z_|_ave_ values approach 0.5, wherein the time required for this process increases with the viscosity. In other words, the directions of the easy axes are gradually randomized at the field-free point. This behavior is also reasonable because the steady magnetic torque vanishes while the Brownian torque continues the randomization in the time corresponding to the Brownian relaxation time.

**Fig 6:**
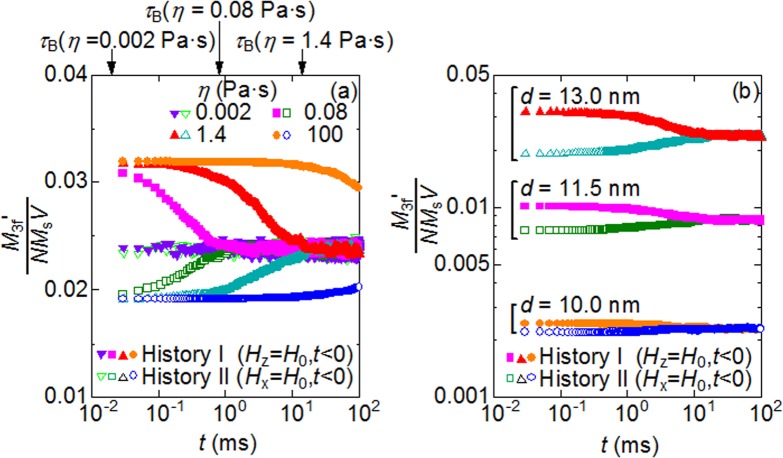
Evolution of *M*_3f^′^_ with easy axes randomization for two field histories. Evolutions of the third-order harmonics of magnetization, *M*_3f^′^_, with randomization of the directions of the easy axes for the field histories I and II (see also Fig. 5). (a) Viscosity-dependence at *d* = 13 nm (b) Size-dependence at *η* = 1.4 Pa s.

At this stage, it is useful to recall the fact discussed earlier, that the magnetization curves of anisotropic superparamagnetic nanoparticles depend upon the orientations of the easy axes. In this context, we can expect that the relaxations of the orientations at the field-free point are accompanied by an evolution of the magnetization curve. Fig. 4(B) shows the results of the simulations for the field histories I and II. We find that the magnetization curve for the field history I gradually approaches from above the curve calculated earlier for the case of randomly-oriented easy axes, while that for the field history II does so from below. With these evolutions, the nonlinearity decreases and increases, respectively. Consequently, *M*_3f^′^_ also varies and reaches a steady magnitude in a time comparable to the Brownian relaxation time, *t* ∼ *τ*_B_, as shown in Fig. 6(A). To sum up, in the case of *d* = 13 nm, the third-order harmonic response of the superparamagnetic nanoparticles at the field-free point apparently depends upon the history of the magnetic field in the time-scale of *τ*_B_, despite the fact that *τ*_N_ = 26 ns is much less than *τ*_B_. This history dependence may lead to another artifact in MPI image if the scanning of the field-free point is faster than the Brownian relaxation. Presently it is unclear how long we should wait for the randomization of the easy axes practically, because there is little knowledge concerning the local environments of magnetic nanoparticles accumulated in tumor tissue. A simple method to avoid this problem is the use of smaller particles, because the history-dependence of *M*_3f^′^_ becomes weak with decreasing the particle size, as shown in Fig. 6(B). The reason for this weak history-dependence is that the initial alignment of the easy axes at *t* = 0 is more randomized in smaller particles because the Brownian torque predominates the magnetic torque in smaller particles. It should be noted, however, that the magnitude of *M*_3f^′^_ steeply declines with decreasing particle size (Fig. 6(B)). In other words, this easy method has a trade-off relation between the high-speed scanning of the field-free point for a rapid diagnosis and the use of larger nanoparticles for an intensified signal. For this reason, new scanning protocols need to be constructed with consideration of the dependence upon the history of the magnetic field.

In the present MPI technique, the detected third-order harmonics of the electromotive force, indicating the existence of hidden tumor fragments, are being analyzed on the assumption that the magnetization curve is expressed by the Langevin function. In other words, the nanoparticles are considered to be completely isotropic. Actual nanoparticles, however, do not satisfy this condition. Therefore, we simulate the magnetic responses of anisotropic magnetic nanoparticles in high-frequency AC magnetic fields with sufficiently large amplitudes, and attempt to clarify the features of the third-order harmonic component, wherein the magnetic/thermal torque-driven rotation of the easy axes as well as the thermally-activated magnetization reversals over magnetic hard planes are incorporated. Consequently, we find a slight delay of the magnetic response to alternations of the magnetic fields when larger nanoparticles with strong signals are employed. In this condition, the third-order harmonic response for the minor loop is negative while that for the major loop is positive. This fact clearly indicates the possibility that the sign of the signal is reversed when the field-free point scans from the body surface to points deep inside the body, although the states of the magnetic nanoparticles are identical. This may cause artifacts in the reconstructed image. We also find that, even in a superparamagnetic state where no hysteresis loops are registered under the AC field, the amplitude of the third harmonic component strongly depends upon the history of the DC bias magnetic field that aligns the easy axes of the nanoparticles. This is due to the fact that the equilibrium magnetization curve for the field applied at the magnetically hard plane is significantly different from the Langevin function and its non-linear component is extremely small. This simulation study finds, therefore, that MPI signals are seriously affected by the inevitable deviations from the Langevin function that exist with actual nanoparticles.

In this study, we paid attention to the fact that each actual nanoparticle is more or less anisotropic, and pointed the possibilities of artifacts in magnetic particle imaging using numerical simulations for individual anisotropic nanoparticles. At this stage, we must mention that aggregated superparamagnetic nanoparticles may be selected for MPI [5]. If the nanoparticles are not magnetically isolated, dipolar interactions between the nanoparticles would play an important role in magnetic responses to AC magnetic fields in rotatable cases [16] as well as in non-rotatable cases [17]. For this reason, in the next stage, numerical simulations considering such interactions are highly desired for further progress in the development of new tracers using aggregated nanoparticles.

## Models

### Actual nanoparticles and local environment

The shapes of actual nanoparticles are not completely spherical, which causes a variation in the amount of surface magnetic charge with the direction of ***e***. This is the shape anisotropy energy, *U*_d_. On the other hand, faceted nanoparticles have surface magnetic anisotropy energy, *U*_s_, because of the breaking of the local symmetry at the surface. In addition, atomic lattices without spherical symmetry induce a crystalline anisotropy energy *U*_c_. Consequently, the total potential energy *U* is given by-*μ*_0_ *M*_s_ *V* ***e*· *H*** *+ U*_d_ *+ U*_c_ + *U*_s_ instead of that given in Eq. (1). Because the details of these anisotropies are still unknown [4], this formula has conventionally been simplified to

$$U(n,\;e,\;H)\;=\;-\mu_{0}M_{\text{s}}V\;e∙H\;-K_{\text{eff}}V{\left(n∙e\right)}^{2}\;,$$ (3)

using a lower-order term as a first approximation, where *K*_eff_ is the effective uniaxial anisotropy constant and ***n*** is the unit vector along the easy axis. Considering iron oxide nanoparticles as a typical example, the reported value of *K*_eff_ varies from paper to paper in a range from 10–30 kJ/m^3^ [4,18,19]. This diversity may arise from the differences of the shape or the crystallinity, but the true reason is still unclear. If we choose the intermediate value of 20 kJ/m^3^, the magnitude of *K*_eff_ *V* and the related quantities for nanoparticles with effective diameters of *d* = (6*V*/π)^1/3^ are calculated and shown in Table 1. Compared with the thermal energy *k*_B_ *T* ∼ 4×10^–21^ J at room temperature, the magnetic anisotropy expressed by the second term is not negligible for nanoparticles in the size range *d* ≥ 10 nm. On the other hand, the calculated Néel relaxation time, ***τ***_N_, indicates that a hysteresis loop should be observed for nanoparticles with *d* = 23 nm when the measurement using a vibrating sample magnetometer takes one hour. Because they have not been customarily regarded as superparamagnetic, we studied the properties of anisotropic nanoparticles in a size range of 10–20 nm diameters. When calculating *M*_s_, the value of 450 kA/m obtained for bulk magnetite was used as is [18], because a difference of *M*_s_ does not seriously affect the results of the simulations. The poly-disperse nature of real nanoparticles was considered as a logarithmic normal distribution of *d* with a standard deviation *σ* = 0.2, where the cutoff is ±2*σ*.

Little is known about the local environments of magnetic nanoparticles accumulated in tumor tissue. If a passive targeted drug delivery system is used, the nanoparticles may be dispersed in intracellular media whose viscosity *η* is not constant, but whose *η* is 1–3 ×10^–3^ Pa s in aqueous domains while the *η* = 8×10^–2^ Pa s in other areas [20]. Surprisingly, there is a report giving the value of *η* = 1.4 Pa∙s [21]. Nanoparticles used in conjunction with tumor-homing peptides or antibodies for active targeting, however, should be anchored to a cell membrane. Recently, in-situ X-ray diffractometry on a single nanoparticle showed that a nanoparticle anchored to a substrate by an antigen-antibody combination or by a protein can slowly rotate on the time scale of milliseconds to seconds [22,23]. Assuming a kind of Brownian mechanism, *η* can be nominally estimated to be roughly 100 Pa s. To sum up, the nanoparticles in tumor tissue are, in many cases, rotatable, although the nominal viscosity is highly sensitive to the accumulated state. Therefore, we incorporated rotations into the simulation in the latter part, where values of *η* of 2×10^−3^, 8×10^−2^, 1.4 and 100 Pa∙s were employed as typical examples.

### Simulation of Néel relaxation

The orientation probability *ρ*(***e***) of the direction of ***e*** is given by a Boltzmann factor exp(−*U*(***n***, ***e*, *H***)/*k*_B_ *T*) using Eq. (3). As indicated by their values for *d* ≥ 10 nm in Table 1, *ρ*(***e***) at ***e*** // ***n*** is higher by more than one digit compared with that at ***e*** ⊥ ***n*** at *H* = 0. Therefore, the direction of ***e*** can be considered to be localized at ***e***_1_ parallel to the easy axis (or the antiparallel direction ***e***_2_). In other words, we can simply assume that *ρ*(***e***_2_) = 1 – *ρ*(***e***_1_). In this case, we can use the two-level approximation that considers thermally-activated reversals between the minima *via* the midway saddle point, ***e***_3_, at the hard plane. The reversal probability from ***e***_1_ to ***e***_2_, *υ*_12_, is given by 0.5*f*_0_ exp[–(*U*(***n***, ***e***_3_, ***H***) – *U*(***n***, ***e***_1_, ***H***))/*k*_B_ *T*], while the backward reversal probability *υ*_21_ is 0.5*f*_0_·exp[–(*U*(***n***, ***e***_3_, ***H***) – *U*(***n***, ***e***_2_, ***H***))/*k*_B_ *T*], where *f*_0_ is the attempt frequency of 10^10^ s^–1^. In this framework, ***τ***_N_ is given by (*υ*_12_ + *υ*_21_) ^–1^. In this work, the time evolution of *ρ*(***e***_1_) was computed using *Δρ*(***e***_1_) = [*υ*_21_ *ρ*(***e***_2_) – *υ*_12_ *ρ*(***e***_1_)]*Δt* for an AC magnetic field (0, 0, *H*_ac ·_sin(2π*f*m·*t*)) with various *H*_ac_ and an *f* of 50 kHz. The time step *Δt* was typically 10^−4^/*f* s, but was shorter when *υ*_12_ *Δt* (or *υ*_21_ *Δt*) became large compared with one. At each step, the magnetization *M*(*t*) was obtained as *NM*_s_ *V* [*ρ*(***e***_1_)***e***_1_·(***H***/*H*) *+ ρ*(***e***_2_)***e***_2_·(***H***/*H*)]. The higher-order harmonics of *M*(*t*) were analyzed using the Fourier series $\sum_{\text{n}}\left[M_{\text{nf}}^{\prime }\text{sin}\left(2\text{n}\pi ft\right)-M_{\text{nf}}^{\prime \prime }\text{cos}\left(2\text{n}\pi ft\right)\right]$.

### Simulation of Brownian relaxation

In this study, we simulated rotations of magnetite nanoparticles smaller than *d* = 13 nm, where *f* was again set at 50 kHz. For these nanoparticles, ***τ***_N_ was shorter than 26 ns, while the switching of the field occurred at every 10 μs at *f* = 50 kHz. In other words, *ρ*(***e***) can be regarded to be always equilibrated in *U*, although the Zeeman energy varies slowly. In this case [24], the magnetic torque on the easy axis ***T***_r_ is given by the average,

$$T_{\text{r}}\;=\;\int \left(-n\;\times \;\partial U\left(n,\;e,\;H\right)/\partial n\right)\rho \left(e\right)\;\text{d}\Omega_{e},$$ (4)

where *Ω*_e_ is the solid angle of ***e***. On the other hand, the frictional torque is expressed as π*ηd*_H_^3^∙***ω*** using the assumption of Newtonian fluid for simplicity, where *d*_H_ is the hydrodynamic diameter of the nanoparticles including surface modification layers and ***ω*** is the angular velocity of rotation. In this simulation, *d*_H_ was assumed to be 30 nm. If the inertia of the nanoparticles is negligible, the torques balance each other as follows:

$$\pi \eta d_{\text{H}}^{3}\omega \;=\;T_{\text{r}}\;+\;\lambda (t),$$ (5)

$$<\lambda_{\text{i}}(t)>=\;0,$$ (6)

$$<\lambda_{\text{i}}\left(t_{1}\right)\lambda_{\text{j}}\left(t_{2}\right)>=\;2k_{\text{B}}T∙\left(\pi \eta d_{\text{H}}^{3}\right)∙\delta_{\text{ij}}∙\delta \left(t_{1}\;-\;t_{2}\right),$$ (7)

where ***λ***(*t*) is the Brownian random torque, i and j indicate the Cartesian vector components, *δ*_ij_ is the Kronecker delta and *δ*(*t*_1_ – *t*_2_) is the Dirac delta function. Using these equations, the time evolution of ***n***(*t*) was computed by the following steps: (i) *ρ*(***e***(*t*)) was calculated by the Boltzmann factor for *U*(***n***, ***e***, ***H***) with the latest ***n***(*t*) and ***H***(*t*), (ii) ***T***_r_ was computed by substituting *ρ*(***e***(*t*)) into Equation (4), (iii) ***ω*** was calculated by substituting ***T***_r_ into Equation (5), and (iv) ***n***(*t*) was finally replaced by ***n***(*t +Δt*) = ***n***(*t*) + [***ω***(*t*) **× *n***(*t*)]*Δt*, where the time step *Δt* was 2**×**10^–4^/*f* = 4 ns. At each step, the magnetization *M*(*t*) was obtained as $NM_{\text{s}}V\;\int \left(e∙H/H\right)\rho \left(e\left(t\right)\right)\;\text{d}\Omega_{\text{e}}$. The higher-order harmonics of *M*(*t*) were analyzed as stated above.

In this study, the scanning of the field-free point was roughly classified into the longitudinal scan and the transversal scan, where the history of the magnetic field in the former case (history I) was modeled as (0, 0, *H*_0_) at—∞ < *t* < 0 and (0, 0, *H*_ac_·sin(2π*f*·*t*)) at *t* > 0, while the history in the latter case (history II) was (*H*_0_, 0, 0) at -∞ < *t* < 0 and (0, 0, *H*_ac_·sin(2π*f*·*t*)) at *t* > 0. The magnitude of a DC bias magnetic field *H*_0_ was set at 100 kA/m, considering the magnetic field required for saturation in Fig. 4 (A), while *H*_ac_ was at 10 kA/m. Because of the sufficiently long period in ***H***_0_, the system could be considered to be equilibrated well at *t* = 0, and the distribution of ***n***(*t* = 0) and ***e***(*t* = 0) should be independent of *η*. For this reason, the rotations of 18000 nanoparticles in an aqueous phase with *η* of 2×10^−3^ Pa s were simulated in ***H***_0_ beforehand, and their steady state was employed as the common initial state of the main simulations for the behavior in the field-free point in various viscous media with *η* values of 2×10^−3^, 8×10^−2^, 1.4 and 100 Pa s at *t* > 0.
